# Augmented reality tumor mapping: eliminating positive margins in oncosurgery

**DOI:** 10.1097/MS9.0000000000004688

**Published:** 2026-01-06

**Authors:** Muhammad Khizar, Muhammad Zaib, Savera Ejaz Ahmed, Hasiba Karimi, Hasibullah Aminpoor

**Affiliations:** a Faculty of Medicine, Georgian American University, Tbilisi, Georgia; b Department of Medicine, Shaheed Mohtarma Benazir Bhutto Medical College, Lyari, Karachi, Pakistan; c Faculty of Medicine, Bezmialem Vakif University, Istanbul, Turkey; d Faculty of Medicine, Kabul University of Medical Sciences “Abu Ali Ibn Sina”, Kabul, Afghanistan

**Keywords:** cancer, medical mapping, oncologic surgery, precision medicine, tumor

## Abstract

Augmented reality (AR) is transforming cancer surgery by combining medical imaging with real-time visualization during operations. Using 3D digital models of tumors, AR allows surgeons to see the precise location and borders of cancers directly on the patient. Early studies in breast, brain, urologic, and skin cancers show that AR improves accuracy and helps surgeons remove tumors more completely and safely. Research from China and South Korea has demonstrated accurate margin identification in skin and breast tumor surgeries, while teams in Italy and Belgium have reported better surgical planning and shorter operation times in prostate and brain tumor procedures. AR is also being used to train surgeons in low-resource settings, improving access to advanced surgical methods worldwide. Positive surgical margins, which are associated with increased local recurrence rates and reduced survival, highlight the critical need for accurate intraoperative tumor visualization across tumor types. Although more clinical trials are needed to confirm its ability to reduce positive margins, AR-guided tumor mapping shows great promise as a standard tool to support safer, more precise oncologic surgery.

*To the Editor,*

Positive surgical margins continue to be a major obstacle in achieving complete cancer removal. When tumor cells remain at the resection edge, the risk of recurrence rises significantly. For example, in breast-conserving surgery, positive margins increase local recurrence rates up to 20%, while in prostatectomy, positive margins can elevate biochemical recurrence risk by 15–25%. In brain tumor resections, residual tumor correlates with shorter progression-free survival. Augmented reality (AR) tumor mapping offers a modern solution by merging preoperative scans with live surgery, projecting 3D images of tumors and nearby vital structures into the surgeon’s view. This technology improves orientation, decision-making, and precision in tumor excision^[1]^.

In neurosurgery, Van Gestel *et al* in Belgium found that AR planning improved incision accuracy and reduced preparation time compared with traditional neuronavigation^[2]^. In Italy, Musi *et al* launched a randomized phase III trial to evaluate whether AR-assisted robot-assisted prostatectomy can lower positive margin rates^[3]^. Similarly, in breast cancer surgery, Ock *et al* in South Korea demonstrated that an AR-based tumor localization system matched the accuracy of 3D-printed guides while allowing greater flexibility during operations^[4]^. In China, Li *et al* combined AR with artificial intelligence to help surgeons identify tumor borders in real time, improving precision in skin cancer excisions^[5]^.

Despite promising results, the practical rollout of AR-guided tumor mapping faces several challenges. These include high initial device costs, the learning curve for surgeons, variability across centers in hardware and software integration, and dependence on high-quality imaging. Resource-limited settings may face additional barriers such as infrastructure, technical support, and cost-effectiveness considerations.

AR is also being used globally to bridge surgical education gaps. Arboleda *et al* reported that remote AR-based mentorship programs now link surgeons in the United States and United Kingdom with trainees in Vietnam and Mozambique, expanding access to expert guidance and modern techniques^[6]^. These developments show AR’s value not only as a surgical tool but also as a global equalizer in healthcare innovation.

Looking ahead, future research should focus on large-scale, multicenter trials to validate AR-guided resections across tumor types. Integration with artificial intelligence, including advances such as the AI AlphaFold model^[7]^ in molecular biology and large language models in clinical decision support, could enhance tumor border prediction and real-time surgical guidance. The development of AI-augmented decision-support systems promises to further improve surgical precision and personalized planning.

As imaging, artificial intelligence, and AR technology continue to advance, tumor mapping is set to play a vital role in achieving margin-negative resections across specialties. Further clinical trials and standardized protocols will be essential to confirm its long-term benefits. These combined efforts will ensure that AR-guided tumor mapping is both clinically effective and widely implementable, while addressing equity and accessibility in global surgical care.

Our work is in line with the TITAN Guidelines on the need for transparency in AI use in healthcare^[8]^.

## Data Availability

Not applicable.
